# Interspecies Avian Brain Chimeras Reveal That Large Brain Size Differences Are Influenced by Cell–Interdependent Processes

**DOI:** 10.1371/journal.pone.0042477

**Published:** 2012-07-30

**Authors:** Chun-Chun Chen, Evan Balaban, Erich D. Jarvis

**Affiliations:** 1 Department of Neurobiology, Howard Hughes Medical Institute, Duke University, Durham, North Carolina, United States of America; 2 Behavioral Neurosciences Program, McGill University, Montreal, Quebec, Canada; Utrecht University, Netherlands

## Abstract

Like humans, birds that exhibit vocal learning have relatively delayed telencephalon maturation, resulting in a disproportionately smaller brain prenatally but enlarged telencephalon in adulthood relative to vocal non-learning birds. To determine if this size difference results from evolutionary changes in cell-autonomous or cell-interdependent developmental processes, we transplanted telencephala from zebra finch donors (a vocal-learning species) into Japanese quail hosts (a vocal non-learning species) during the early neural tube stage (day 2 of incubation), and harvested the chimeras at later embryonic stages (between 9–12 days of incubation). The donor and host tissues fused well with each other, with known major fiber pathways connecting the zebra finch and quail parts of the brain. However, the overall sizes of chimeric finch telencephala were larger than non-transplanted finch telencephala at the same developmental stages, even though the proportional sizes of telencephalic subregions and fiber tracts were similar to normal finches. There were no significant changes in the size of chimeric quail host midbrains, even though they were innervated by the physically smaller zebra finch brain, including the smaller retinae of the finch eyes. Chimeric zebra finch telencephala had a decreased cell density relative to normal finches. However, cell nucleus size differences between each species were maintained as in normal birds. These results suggest that telencephalic size development is partially cell-interdependent, and that the mechanisms controlling the size of different brain regions may be functionally independent.

## Introduction

Comparative analyses have suggested that relative brain size differences among species correlate with behavioral complexity [1], [2], [3]. Specifically, proportional enlargements of particular brain regions or pathways are thought to give rise to enhanced behavioral capacities for abilities that are influenced by those brain regions. Such modifications of brain structure have generally been related to broad patterns of evolutionary specialization within particular vertebrate groups, such as hippocampal size and spatial memory in food-storing birds, tectal size and visual acuity differences among avian species, and cortical regional size differences and the complexity of social behaviors in both primate and fish species [2], [4], [5], [6], [7], [8], [9], [10], [11], [12].

Songbirds, parrots, and humans are well known for their vocal learning abilities [13], [14], [15], [16]. These abilities depend on a set of interconnected brain nuclei located mainly within the telencephalon [17], [18], [19]. All three groups have also evolved a disproportionately large adult telencephalon relative to the average vertebrate, and this increased size is thought to be due at least in part to additional circuitry responsible for song and speech learning, and/or to an associated increase in size of other brain regions involved in communicative, social and cognitive abilities [2], [6], [20], [21].

Expanded telencephalon sizes have also been correlated with differences in brain and body development [22]. All vocal learning bird species (and humans) are altricial and exhibit a greater amount of post-natal brain growth, as compared to precocial species, such as chickens, quails and ducks [2], [10], [20], [23], [24], [25]. This post-natal growth may be related to an expanded population of progenitor cells in the embryonic subventricular zone that gives rise to mature brain cells [10], [26], [27]. Concordant with this idea, vocal learning avian species have a relatively larger pool of cells in the telencephalic ventricular zone during development, and have a relatively elongated period of neurogenesis between hatching and sexual maturity, associated with a delayed enlargement of the telencephala compared to many other species [10], [28], [29], [30]. This extended period of cell proliferation is thought to endow altricial species, including vocal learners, with a greater capacity for cultural transmission of behavior via interactions with parents and peers. This does not mean that all altricial species are vocal learners, but rather suggests that altricial brain development could be a precondition for evolving more complex behaviors, including culturally transmitted behaviors [10].

Here we asked whether this developmental difference in telencephalon enlargement between a precocial species and an altricial species is a fully cell-autonomous process or includes cell-interdependent processes. To address this question, we performed forebrain transplantation surgery between the altricial vocal learning zebra finches and the precocial vocal non-learning Japanese quail. We substituted embryonic telencephala from zebra finch donors into Japanese quail hosts (from whom the same region had been removed) at the neural tube stage, and harvested the chimeric embryos more than one week after surgery. The donor and host portions of these chimeric brains successfully fused, and we found that the quail brain environment induced an accelerated enlargement of the transplanted zebra finch telencephalon with an associated increase in cell number and decrease in cell density. We propose that a partly cell-interdependent process influenced by developmental factors from outside of the forebrain contributes to the development of forebrain size differences among these different species.

## Materials and Methods

### Ethics statement

All work was performed in compliance with the animal care and use guidelines of the Duke University Institutional Animal Care and Use Committee. The animal protocol was approved by the same committee (Protocol A133-11-05).

### Animals

Adult zebra finches (*Taeniopygia guttata*; n = 120) were housed in an indoor aviary with three mating pairs per cage. Each week, 1–2 eggs were harvested from each cage. Adult Japanese quails (*Coturnix coturnix japonica*; n = 48) were housed in commercial pens (one male and one female per pen), and 1 egg was harvested from each pair every 1–2 days. Both Japanese quail eggs and zebra finch eggs were collected within 3 hours after the females laid the eggs.

### *In ovo* surgery and electroporation

**Figure 1:**
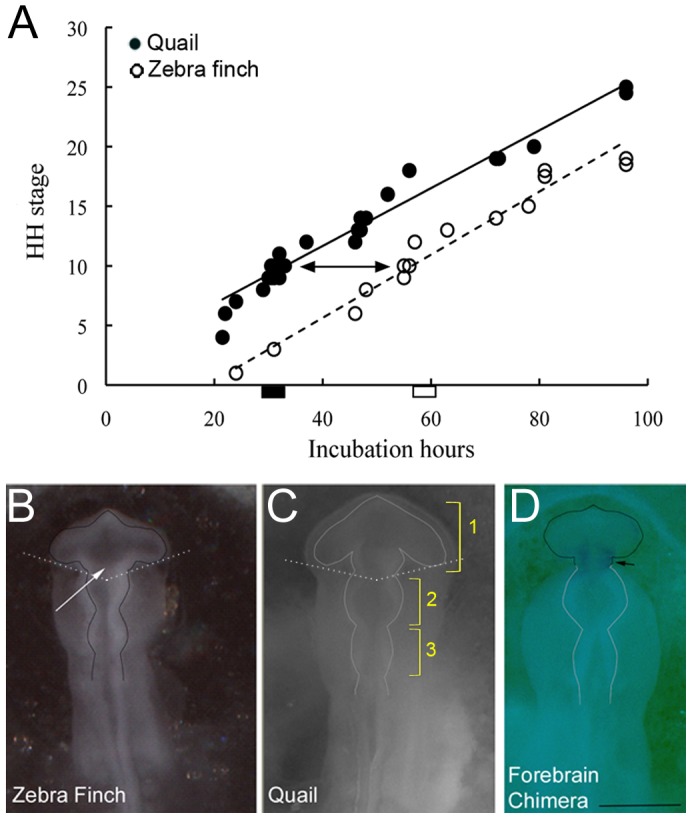
The procedure for *in ovo* transplantation surgery. (A) Graph showing the relationship of developmental stages and incubation times in zebra finch (white circles; n = 39; dashed line) and quail embryos (black circles; n = 50; solid line). Lines = linear regression. Quail and zebra finch embryos were taken for surgery between HH stages 8 to 13 (arrows). This surgery time window is 30–36 hours (black bar) for quail and 55–60 hours (white bar) for zebra finch eggs. (B) Dorsal view of the neural tube in zebra finch donor before transplantation surgery. (C) Quail host before surgery. (D) Chimera immediately after surgery, labeled with fast green in the finch graft. The finch prosencephalon is outlined with a black line and the quail with a white line. At this stage, the anterior neural tube forms three major parts: 1) prosencephalon (forebrain), 2) mesencephalon (midbrain), and 3) rhombencephalon (hindbrain). White arrow, injection location of the GFP plasmid; dashed white line, location for cutting out the transplanted prosencephalon; black arrow, boundary between zebra finch graft and quail host tissue after transplantation. Scale bar = 250 µm.

To perform avian *in ovo* surgeries, we followed a protocol for chicken-quail chimeras [31] with modifications (described below). We incubated quail eggs at 37.5–37.7°C and 45–50% humidity for 30–36 hours and zebra finch eggs at 37.5–37.7°C and 45–50% humidity for 57–63 hours in a commercial egg incubator (Brinsea, USA). This incubation timing ensured that both quail and zebra finch embryos were at similar developmental Hamburger-Hamilton (HH) stages [32], which we calculated by counting the number of somites (Figure 1A). Embryonic surgeries were then conducted under sterile conditions under a dissection microscope (Leica M205C, Germany) at 37°C and 30–35% humidity. Under our conditions, about a 25-hour window of time separated the more rapid embryonic development of the quail and the slower development of the zebra finch early embryos (Figure 1A). We thus performed surgeries at around the 10-somite stage, range HH stages 8–13 (Figure 1A), because the position of the neural tube was still relatively straight and blood vessels had not yet formed.

#### Treatment of zebra finch donors

After 57–63 hours of incubation, the surface of the eggshells was cleaned with 70% alcohol and a large opening made with sterile forceps. Thereafter, zebra finch cells in the posterior prosencephalon (primordial posterior telencephalon and thalamus) were labeled by injection of a CAG (CMV early enhance/chicken β actin)-GFP plasmid (5 ug/ul) suspended in fast green dye (Invitrogen, USA). The GFP plasmid mixture was electroporated laterally in the zebra finch neural tube using 25 V, 5 pulses, 50 ms duration and 100 ms interpulse interval (BTX350, Harvard instrument, USA). After electroporation, the donor blastoderm was cut out from the egg with iridectomy scissors and stainless steel microscalpels, transferred into a sterile plastic Petri dish coated with a black colored base [31] containing sterile phosphate buffered saline solution (PBS; pH = 7.4; Figure 1B). The zebra finch prosencephalon was dissected out and transferred into a dish of fresh sterile PBS, and any attached notochord left behind was carefully removed. This electroporation approach allowed for cell fate determination while avoiding false positive labeling, unlike that seen with more leaky lipophilic tracer labeling [31].

#### Treatment of quail hosts

After cleaning the quail eggs with 70% ethanol, ∼0.3 ml of albumin was removed with a syringe needle punctured into the sharp end of the egg, to lower the embryo inside the shell. We then laid the egg on its side, and carefully opened an approximately 1-cm^2^ window of shell on the upper side, using a curved surgical scissor. To visualize the embryo with higher contrast, we used sterile blue food-coloring dye (FD&C Blue No. 2), diluted 1∶1 in sterile PBS, injected under the blastoderm with a glass micropipette. The quail prosencephalon was excised from the host embryo using stainless steel microscalpels *in ovo* and removed using a glass micropipette (Figure 1C). The prosencephalon of the zebra finch donor was transferred to the host embryo and placed in the groove produced by the excision, in the normal rostro-caudal and dorso-ventral orientation (Figure 1D). After transplantation, the surgery window of the host quail eggs was sealed with sterile surgical tape (3 M, USA) and melted paraffin surrounding the window edge. For a sham control group, quail eggs were windowed, injected with blue food-coloring dye, and sealed as the chimera group. We incubated the surgically treated quail and zebra finch chimeric embryos at 37.5–37.7°C and 45–50% humidity until embryonic day 9, 12 or 16. Under these incubation conditions, normal zebra finch eggs hatch around embryonic day 13–14 and normal quail eggs around embryonic day 17–18.

### Embryo collection and tissue treatment

We collected all surviving embryos. At embryonic day 9, the skin surrounding the skull was removed. At embryonic days 12 and 16, the skin with feathers was removed from zebra finch heads, and in addition, the skull was removed from the quail and chimera heads. Harvested embryonic heads and brains were fixed in 4% paraformaldehyde in PBS (pH = 7.4) for 3–7 days, cryoprotected in 30% sucrose in PBS, and frozen in Tissue-tek O.C.T in a block mold (Sakura, Japan), on top of a dry ice and 100% ethanol mixture. The heads and brains were sectioned sagittally at −18–20°C on a cryostat in 5 alternative series, at 14–18 µm thicknesses, mounted onto Superfrost plus slides (Fisher Scientific, USA) and stored at −80°C. Alternatively, to process tissues for paraffin sectioning, six embryos were fixed in 4% paraformaldehyde in PBS (pH = 7.4) for 3–7 days, dehydrated in 30%, 70% and 100% ethanol, and embedded in paraffin wax (Fisher Scientific, USA). The paraffin-embedded embryos were sliced sagittally at 10 µm thickness on a microtome (Lecia RM2025, Germany), mounted on slides and stored at room temperature.

### Nucleolus marker staining and immunocytochemistry

We initially attempted to see if antibodies that distinguish neurons from glia (such as NeuN) could provide acceptable results for measuring the boundaries of the transplanted brain regions and for conducting cell counts. However, these types of staining were notably inferior to the techniques we detail below. This led us use techniques that allowed us to identify graft boundaries and count cells in an unambiguous manner.

We found that we could distinguish zebra finch cells from quail host cells in interspecies chimeras by staining zebra finch and quail embryos with two nucleolus markers, hematoxylin and eosin (HE) and DAPI, and the quail cell marker antibody QCPN (QCPN antibody; [33]). For HE staining, paraffin sections of avian embryos on Superfrost plus glass slides (Fisher Scientific, USA) were deparaffinized in xylene, rehydrated in a diluted ethanol series, and stained in Harris hematoxylin solution. After washing, the sections were counterstained with eosin-phloxine solution, dehydrated, delipidized and coverslipped with Permount medium (Fisher Scientific, USA). For QCPN and DAPI double staining, sections were stained first with a mouse anti-quail QCPN cell antibody (Hybridoma bank, Iowa) diluted 1∶1 in the blocking solution (3% normal goat serum in PBS), reacted with a secondary fluorescent Alexa 594 conjugated antibody (Molecular Probes, OR) diluted 1∶200 in PBS and finally counterstained with DAPI (Vector Labs).

For double and triple labeling of GFP with other markers, we applied sequential immunocytochemisty staining on fixed frozen sections. Sections were removed from the −80°C freezer, rehydrated in PBS, blocked in blocking solution for 1 hour, and incubated overnight at 4°C with a primary rabbit anti-GFP antibody (Invitrogen, USA) diluted 1∶200 in the blocking solution. They were then washed with PBS, and reacted overnight at 4°C with a secondary fluorescent Alexa 488 conjugated antibody (Molecular Probes, OR) diluted 1∶200 in PBS. They were then washed with PBS, and reacted with the QCPN primary antibody or with a mouse anti-quail neural fiber antibody (QN; [34]) diluted 1∶3, and then reacted with the secondary fluorescent Alexa 594 conjugated antibody, diluted 1∶200 in PBS. The QN antibody was kindly provided by Dr. Tanaka, Kumamoto University. The sections were counterstained with DAPI. To detect neural fibers in both species, adjacent sections were stained under a similar protocol with a rabbit anti-microtubule-associated protein type 2 (MAP2) primary antibody (Millipore, MA) diluted 1∶200, and reacted with a secondary fluorescent Alexa 594 conjugated antibody (Molecular Probes, OR), and counterstained with DAPI. For measuring the optical density of QN staining, we stained both experimental and control sections at the same time with the same procedure to eliminate any experimental batch effects. All photos of QN stained sections were carefully taken under the same microscopy settings. We used the Photoshop 7 (Adobe, CA) histogram function to measure QN optical density and normalized it using the background optical density without tissue on the slide.

### Analyses and Statistics

To determine brain boundaries in embryos, we used DAPI staining of chimeras and in-situ hybridizations applied to sections generated for another project, which had been hybridized with genes (such as FoxP1, CoupTF2, Lxh9, and Dlx6) that define brain regional boundaries (such as mesopallium, nidopallum, arcopallium, and striatum respectively; see abbreviations in Table 1) to help us confirm the brain region localizations reported here (Chen et al, in preparation). In this study, DAPI staining of all samples provided clear morphological boundaries for brain area, and clear visualization of cell nuclei. Brain sizes were measured by systematically sampling every ten sections, estimating the total volume from the surface areas and thickness of the sections. Cell numbers and nucleus sizes were quantified stereologically by systematic random sampling using the Stereo Investigator system (version 6, Microbrightfield, Burlington, VT). Cells containing a strong DAPI signal in the nucleus with a clear edge at the nuclear envelope were counted and measured for nucleus size. We sampled the cells at more than 20 sampling sites per section, and the thickness of the section was empirically determined using the Stereo Investigator system (between 14–18 µm). Each counting frame contained 0 to 5 cells (counting grid of 5 µm). The coefficient of error of each sampled section was less than 8% (usually 2%–4%). Cell density was automatically calculated from sampled cell numbers and area volumes by the Stereo Investigator system. If raw data met the assumptions of normality (Shapiro-Wilk Test) and equal variance tests, then a parametric one-way ANOVA was performed. If not, then a nonparametric Kruskal–Wallis one-way ANOVA was performed. If they revealed significance, Tukey's post-hoc pair wise comparisons were performed for the parametric ANOVA, or Dunn's method was performed for the non-parametric ANOVA [35]. For the QN optical density analyses, if the data passed normality and equal variance tests, a parametric t-test was applied, and if not a Mann-Whitney rank sum test was applied. All statistical tests were performed using Sigma Stat 3.1 and Sigma Plot 12 (Systat Software Inc., San Jose, CA). Data are expressed as means ± standard errors (S.E.M.). The significance value was set at *p*<0.05, two-tailed.

**Table 1:** Abbreviations of brain areas.

A	arcopallium
ac	anterior commissure
AEP	anterior entopeduncular
aT	anterior thalamus
C	cerebellum
DLM	the medial portion of the dorsolateral nucleus of thalamus.
H	hyperpallium
Hab	habenula
Hp	hippocampus
HT	hypothalamus
lfb	lateral forebrain bundle
LSt	lateral striatum
M	mesopallium
Mes	mesencephalon
N	nidopallium
Nc	caudal Nidopallium
Och	optic chiasm
opt	optic tract
Os	optic stem
OT	optic tectum
P	pallidum
POA	preoptic area
pT	posterior thalamus
R	retina
SC	spinal cord
Se	septum
SGC	stratum griseum centrale
St	stratum
T	telencephalon
TH	thalamus
v	ventricle
VT	ventral thalamus
VZ	ventricular zone

## Results

Zebra finches and quails are at distal ends of the neoaves phylogenetic tree, separated by an estimated divergence time of 65 million years or more [36]. They differ both in the duration of their incubation period and their size at birth (quails have a longer incubation period and a much larger body size). Knowledge of the chronology of development in each species is a prerequisite to choosing the best stage for transplantation. Following the standard approach for early chicken embryos [32], [37], we used the number of somites for each species under our incubation conditions to calculate comparative developmental stages (Figure 1A). After mastering the surgical techniques and incubation conditions, we were able to keep chimeric embryos alive up until embryonic day 16 (ED16), one day before the quail host hatching date. None of our chimeric animals hatched (n = 100 cases), nor did they in the small number of cases where attempts were made to aid their hatching. Our surgical-windowed control quail embryos did hatch. Under our incubation conditions, Japanese quail eggs hatched at 17 days of incubation and zebra finch eggs hatched at 13 days. Extrapolating from the early developmental curves, ED16 in quail chronology would be similar to post-hatching day 3 for zebra finches. Thus, we were able to obtain embryos whose brains should have been at post-hatching stages according to a putative intrinsic zebra finch developmental schedule, and could therefore address questions about brain size differences in the developing embryos.

### Zebra finch donor telencephalon fused with the quail host brain

**Figure 2:**
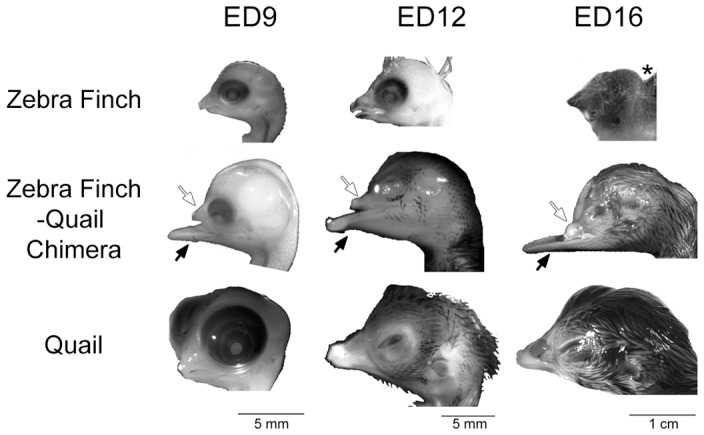
Appearance of the zebra finch, chimera, and quail embryo heads at three developmental stages. Upper row, lateral views of zebra finch embryos and post hatch day 3 animal. *: post hatch day 3 zebra finch is the age most equivalent to embryonic day 16 (ED16) quail. Middle row, zebra finch-quail forebrain chimeras. Bottom row, quail embryos. The eye, forehead, and upper beak of chimeras (white arrows) are derived from zebra finch graft during the surgery, whereas the bottom beak, hind head and necks of chimeras (black arrows) are from quail host.

**Figure 3:**
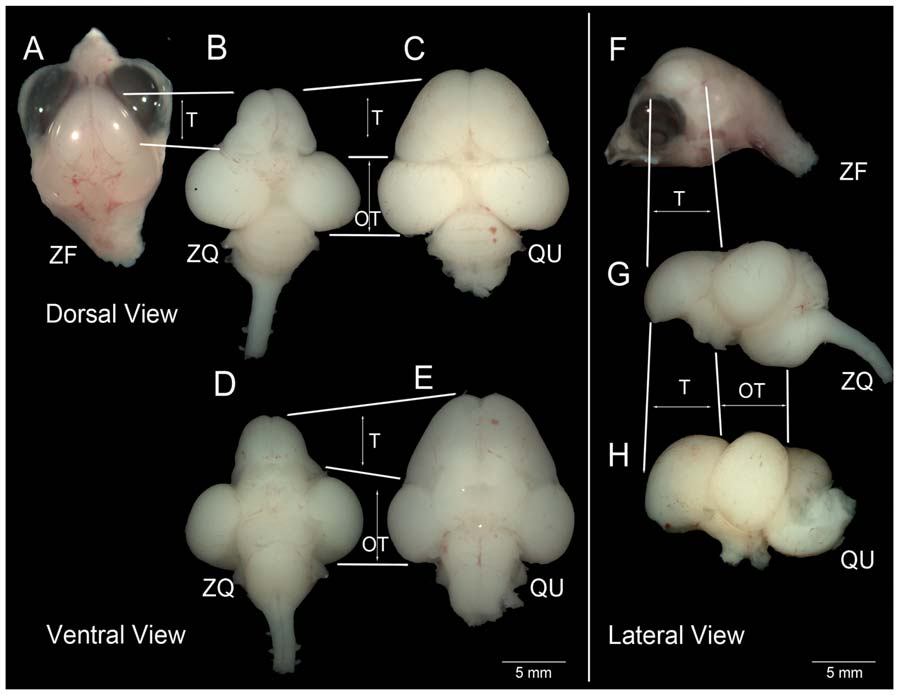
Three views of zebra finch (ZF), chimera (ZQ), and quail (QU) brains at ED12. Dorsal (A–C), ventral (D–E), and lateral (F–G) views of whole brain morphology of each of the three groups, showing that the ZQ chimera is intact and well connected between the grafted forebrain and host brain. The zebra finch brain was left inside the thin skull, as removing it as this age is very delicate and the brain was easily destroyed by adhering to the thin skull. This was not the case for the quail and chimera heads. Lines designate subdivision boundaries. T, telencephalon; OT, optic tectum.

We harvested chimeric embryos at ED9, 12 and 16 using the quail chronology of development and found that they exhibited many characteristics of normal body development. The heads of the zebra finch-quail forebrain chimeras successfully fused, and showed zebra finch characteristics in the anterior dorsal forehead, including the feathers, skin, eyes and beak (Figure 2; white arrows). The anterior ventral parts of finch-quail forehead, including the bottom beak and tongue (Figure 2; black arrows) and the rest of head/body were similar to quail embryos at comparable developmental stages. Inside the skull, the zebra finch telencephalon was well attached to the rest of the host brain at its dorsal, lateral, and ventral aspects (Figure 3). The grafted zebra finch retinae (a derivative of the prosencephalon) were also well connected onto the host optic tecta via the optic nerves projecting from the finch eyes (Figure S1). In all successfully grafted forebrains studied in detail (n = 7), the chimeric zebra finch telencephalon was noticeably larger than normal zebra finch telencephala (Figure 3A vs. B; F vs. G), but still noticeably smaller than normal sham control quail telencephala (Figure 3B vs. C; D vs. E; G vs. H). The remainder of the chimeric brain was morphologically similar to a normal quail brain (Figure 3).

### Zebra finch and quail portions of the brain

**Figure 4:**
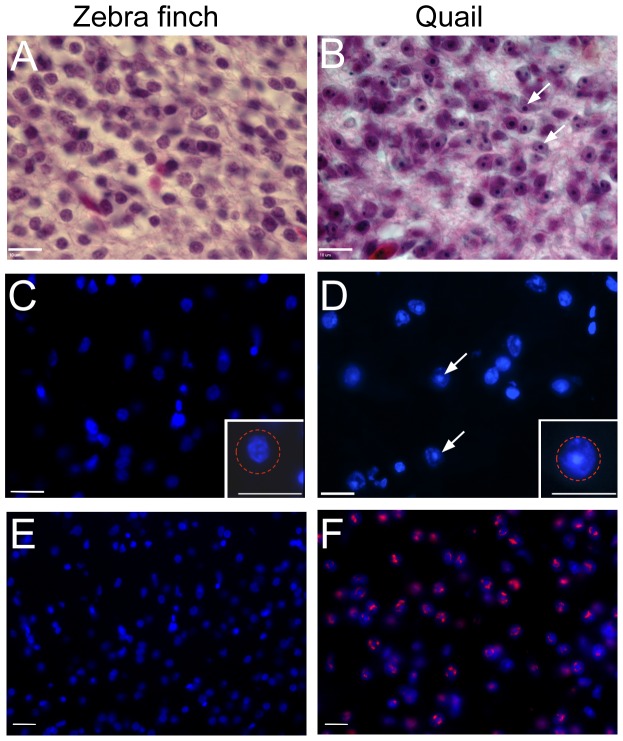
Cell histology differences between quail and zebra finch using three staining methods. (A–B) Hematoxylin and eosin (HE) staining of nuclei in normal zebra finch (A) and quail (B) brain tissue. Arrows point to nucleoli, and show the condensed heterochromatin in the center of the nucleus associated with the nucleolus of the quail cells. (C–D) DAPI staining (blue) in zebra finch (C) and quail (D). Arrows point to nucleoli in the quail cells. Higher magnification of an example of a single zebra finch and quail cell is in the right bottom corner inset of C and D, respectively. The red circle in C is identical in size to the red circle in D, illustrating the nucleus size difference between species. QCPN, quail-specific antibody stains only quail cells (red in F), not zebra finch cells (E) counter stained with DAPI (blue). Scale bar = 10 µm.

To distinguish zebra finch and quail cells in the interspecies chimeric embryos, we applied HE and DAPI nuclear staining, together with the QCPN antibody that recognizes quail cells [33]. Some species of birds, such as the quail, have deeply HE stained nucleoli inside their cells ([38]; Figure 4B), a characteristic that we found was not present in zebra finch cells (Figure 4A). Likewise, in DAPI-stained material, the quail nucleoli were brightly fluorescent (Figure 4D) but zebra finch nucleoli were not (Figure 4C). DAPI staining also revealed that quail nucleus sizes were larger than in zebra finches (Figure 4, inset). The QCPN antibody did not mark any zebra finch cells (Figure 4E), but labeled quail cells (Figure 4F). Thus, we had three independent methods for distinguishing zebra finch and quail cells.

**Figure 5:**
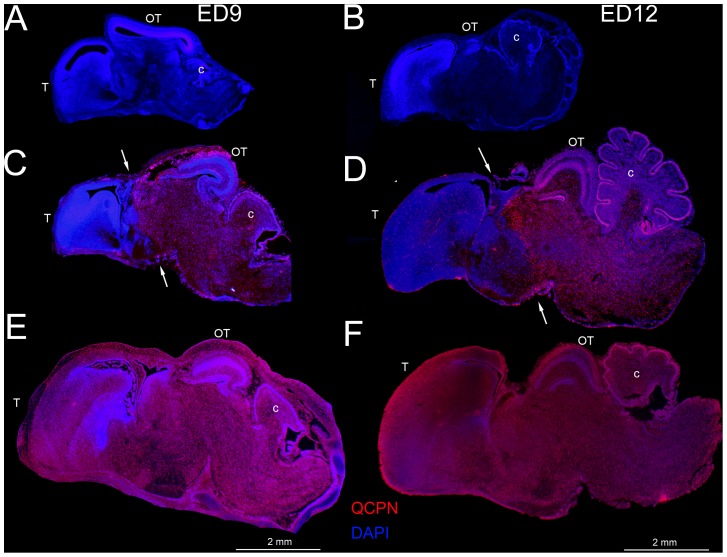
Sagittal sections of zebra finch, chimera and quail embryonic brain stained with the QCPN antibody. QCPN is red and DAPI is blue. (A and B) Stained brain sections of zebra finch at ED9 (left column) and ED12 (right column). (C and D) Stained chimera sections at both ages. White arrows point to the fused boundary of zebra finch graft and quail host between the thalamus and midbrain. (E and F) Stained quail brain sections at both ages.

**Figure 6:**
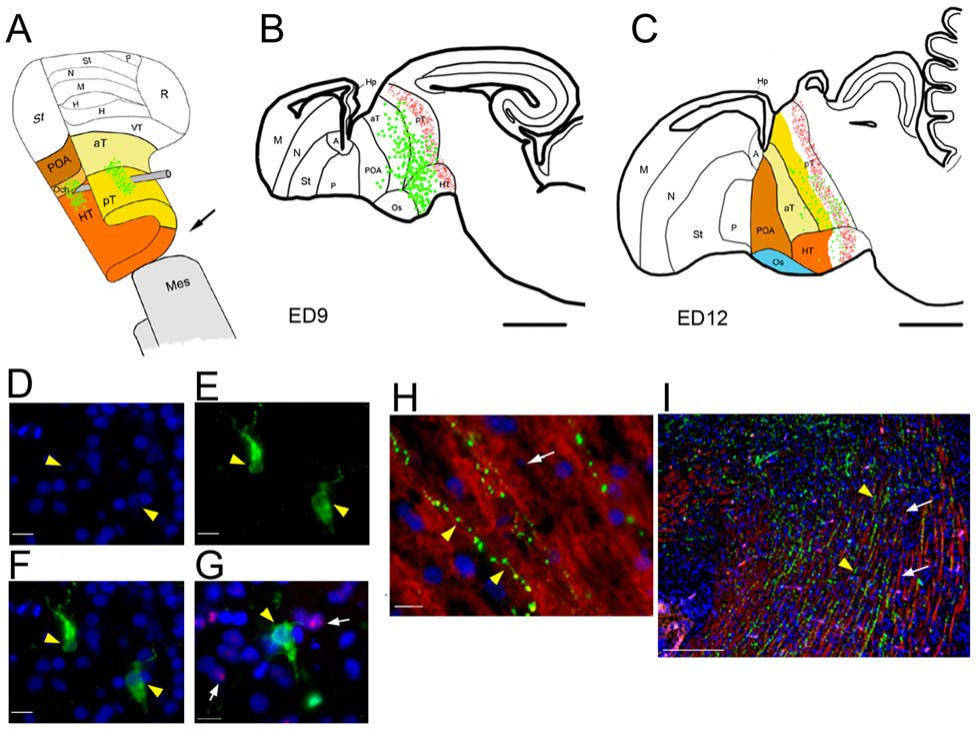
Localization of finch GFP positive cells in the chimera. (A) 3D diagram of the zebra finch neural tube, referring to the chicken cell fate map [39] showing the GFP plasmid injection location (green) in the zebra finch and cutting edge during the transplantation. (B–C) GFP positive cells (green dots) and the boundary between the zebra finch and quail tissue (red dots) are labeled on the sagittal brain contour of chimeras at ED9 (B) and ED12 (C). (D–F) Images of the same section from the chimeric thalamus showing DAPI only, GFP positive cells, and a merging of the two images, respectively. GFP positive cells do not have condensed heterochromatin in the nucleolus, confirming that they are zebra finch cells. (G) Image showing the fusion area in the thalamus region labeled with a zebra finch GFP positive cell (without condensed stained nucleolus; yellow arrowhead) intermingled with QCPN positive quail cells (with a condensed stained nucleolus; white arrows). (H–I) GFP positive cell fibers (green; yellow arrowheads) do not overlap with quail neuronal fibers that stain with the quail neuronal marker QN (red; white arrows). Scale bar = 1 mm in B–C, 10 µm in D–H, and 250 µm in I.

Using this approach, in the chimeras we found zebra finch cells in the telencephala, and quail cells in the posterior thalamus, posterior hypothalamus, tectum, cerebellum, medulla and spinal cord (Figure 5). The boundary between zebra finch and quail cells was found between the thalamic and midbrain areas. Within this boundary, we noted an intermingling of zebra finch and quail cells (Figure 6).

### Zebra finch neurons project into the quail host brain

**Figure 7:**
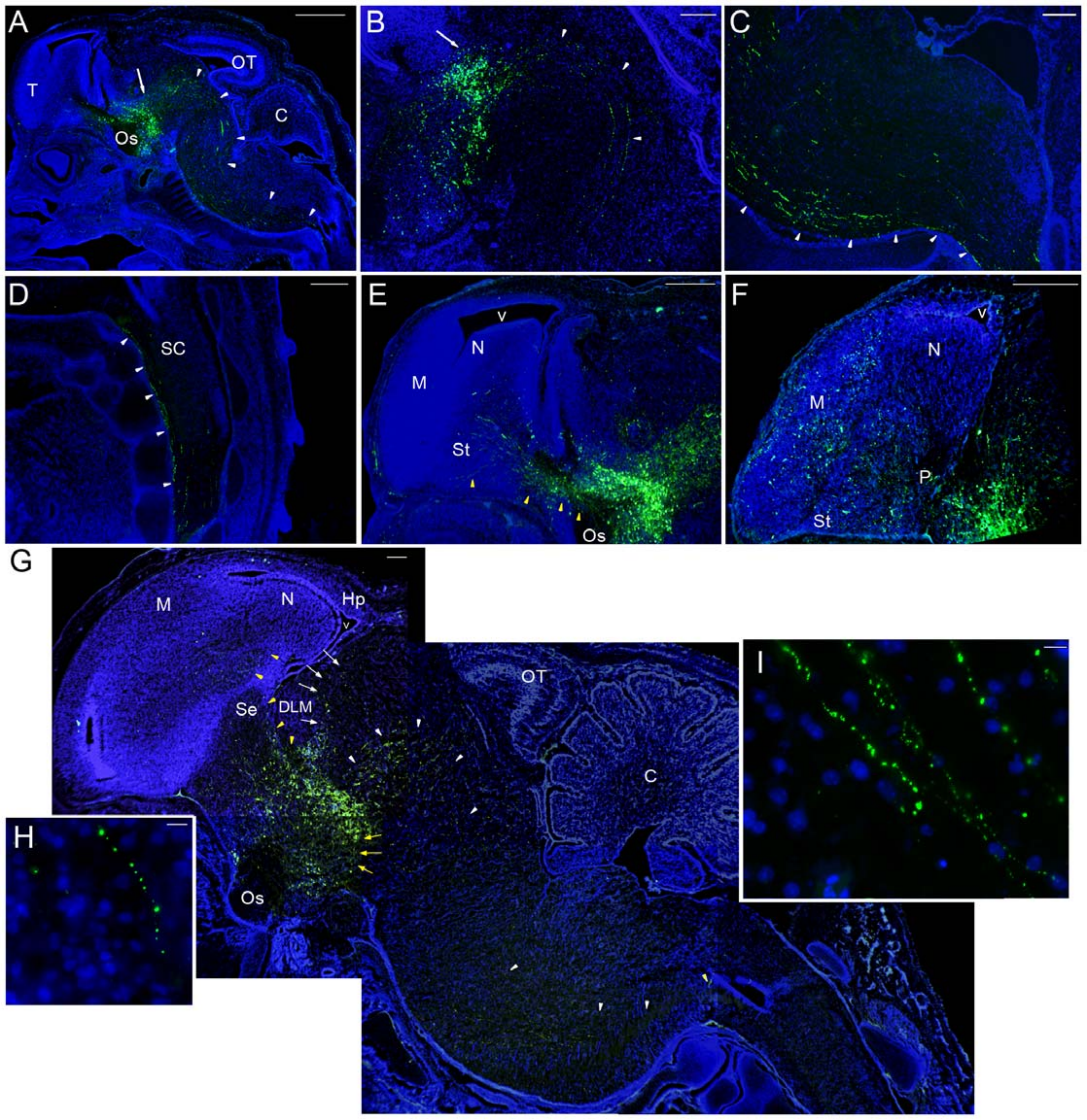
Zebra finch GFP positive cells project into chimeric quail hindbrain and zebra finch forebrain. Embryos are shown with GFP-electroporated zebra finch cells stained with a GFP antibody (green), and all tissue counterstained with DAPI (blue). (A) Chimera brain section at low magnification illustrates GFP stained cells (white arrows) located in the thalamus and GFP positive fibers (white arrowheads) in the spinal cord. (B) Chimera sections in the midbrain area showing the GFP positive fiber tract (thalamic-spinal cord tract) from the thalamic neurons. (C) GFP positive fibers in the quail medulla. (D) GFP positive fibers in the quail spinal cord at higher magnification. (E) GFP positive fibers (yellow arrowheads) in the zebra finch telencephalon passing through the lateral forebrain bundle. (F) GFP positive fibers in the telencephalon, including the pallidum, stratum, nidopallium, and mesopallium. (G) Montage of ED12 chimera brain sections shows the four main tracts of GFP-stained cell fibers: thalamic-spinal cord tract (white arrowheads), thalamic-forebrain tract (yellow arrowheads), thalamic-hypothalamus tract (yellow arrows), and thalamic-habenula tract (white arrows). (H) High magnification view showing GFP positive fibers in finch tissue. (I) High magnification in quail tissue. Panels (A–E) are at ED 9 and (F–I) at ED12. Scale bars = 1 mm in A, 250 µm in B–D, 500 µm in E–F, 200 µm in G and 10 µm in H–I.

To determine whether zebra finch neurons send projections into quail parts of the brain, we analyzed chimeras where the zebra finch cells were electroporated with GFP plasmids in the posterior part of the transplanted neural tube, rostral to the back edge of the graft (Figure 6A). At later developmental stages, GFP-labeled cells could be found just rostral to the boundary between the zebra finch graft and the quail host (Figure 6B–C). Based on DAPI and QCPN staining, all of the GFP positive cells in the chimeras were from zebra finches (Figure 6D–G). The GFP-positive cell bodies were located in the posterior thalamus and hypothalamic areas (Figure 7A), similar to the cell fate of this region of the neural tube in chickens [39]. GFP-positive fibers did not stain with the quail-specific neuron fiber antibody (QN), indicating that the GFP-labeled fibers were also from the zebra finch graft (Figure 6H–I). The finch GFP-positive fibers innervated quail host areas, including the midbrain (Figure 7B), medulla (Figure 7C), and even spinal cord (Figure 7D). The finch GFP positive thalamic fibers also projected into the zebra finch graft telencephalic areas, including the pallidum, striatum, and nidopallium (Figure 7E), along the known telencephalic and thalamic fiber pathways generally seen in birds (Figure 7G; [40], [41]). This indicates that host and donor contributions successfully fused with each other, and that major fiber projection pathways formed normally both within and across the grafted regions.

### Quail neurons project into the finch donor brain

**Figure 8:**
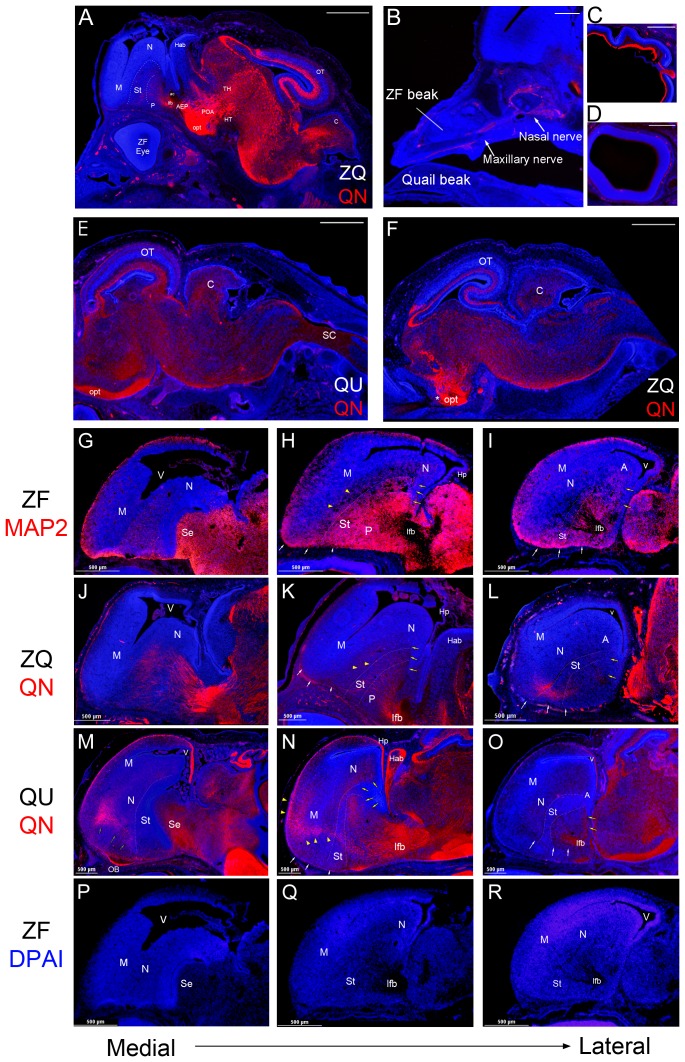
Quail neuronal fibers project into quail hindbrain and zebra finch forebrain. Neuronal fibers are stained with QN or MAP2 antibodies (red) and the cell bodies are stained with DAPI (blue). (A) General distribution of quail neuronal QN stained fibers in a ZQ chimera at ED9. Scale bar = 1 mm. (B) The quail neurons innervate the peripheral tissue of the zebra finch head graft. (C–D) Quail optic ganglion neuron fibers in quail (C), but not in zebra finch retina of chimeras (D). (E–F) Distribution of quail neuronal fibers in quail brainstem (E) is similar to the distribution in chimeras (F) at ED9. However, the optic fibers from zebra finch graft eyes (*) are not stained by QN in chimeras. (G–I) Neuronal fibers patterns of forebrain stained by MAP2 in zebra finch. (J–L) Stained by QN in chimeras. (M–O) Stained by QN in quails. (P–R) Stained by QN in zebra finches. Each column shows a similar section level from medial (left) to lateral (right). The fiber staining of all brain sections shows two similar tracts from pallidum to caudal nidopallium (yellow arrows) and to anterior ventral mesopallium (white arrows) in zebra finch (H–I), chimera (K–L) and quail (N–O). In quail brain sections (M–N), the neuronal fiber tract (yellow arrowheads) is from pallidum through nidopallium to anterior dorsal mesopallium. The QN fibers were strongly stained in the quail hippocampus (N), not in the chimera (K). In chimera (K) and zebra finch (H) sections, the neuronal fiber tract (yellow arrowheads) only extends to the nidopallium. (P–R) The zebra finch forebrain did not stain with the QN antibody, as expected. Scale bar = 1 mm in A, C, and D; 500 µm in B and D.

To determine whether quail neurons sent projections into zebra finch parts of the brain, we used MAP2 to locate all neural fibers in both zebra finch and quails and QN to specifically locate quail neural fibers. In the chimeras, QN-positive quail fibers were detected throughout the nervous system (Figure 8A). For example, in the peripheral nervous system of the chimeras, the finch nasal and beak areas were innervated by the quail cranial nerves (Figure 8B). The optic nerves were labeled by QN staining in normal quail embryos (Figure 8C), but not in the chimeras (Figure 8D), consistent with the finding that the chimeric optic nerves were derived from the zebra finch eyes. Except for the continuation of the optic nerve fibers (Figure 8F, * marker), the QN staining patterns in the midbrain and hindbrain were also highly similar between normal quail embryos (Figure 8E) and chimeras (Figure 8F).

**Figure 9:**
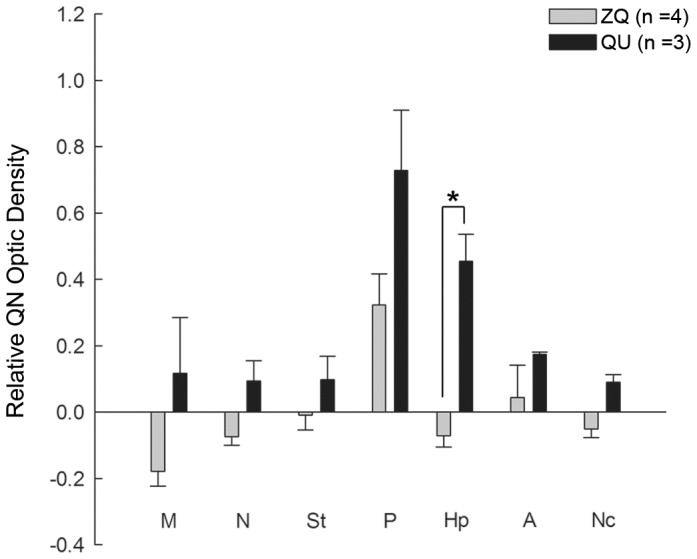
Relative QN staining optical density in the subregions of the forebrains at ED9. QN optic densities in the subregions were normalized by the QN staining density in the tectum. QN optical density of the measured forebrain areas in quail embryos was higher than the optical density in chimera embryos (*p*<0.001; nonparametric two way ANOVA; species×selected brain region). *: *p*<0.004; t-test in the striatum, pallidum and hippocampus and Mann-Whitney rank sum test in the rest of the areas (n = 3 ZQ and 3 QU). Error bars S.E.M.

In the forebrain, the MAP2 antibody labeled fibers in the pallidum, striatum, caudal nidopallium, and anterior ventral mesopallium in zebra finches (Figure 8G–I). In the chimeras, we found QN stained quail fibers in the zebra finch forebrain, including in the pallidum, striatum, caudal nidopallium, and anterior ventral mesopallium (Figure 8G–O). However, the density of QN quail fibers in chimeras was much less in the anterior dorsal mesopallium and hippocampus than that seen in normal quail brains (Figure 8K and 8N). We confirmed this difference in the hippocampus by quantifying the staining density of quail QN fibers normalized to the staining density in the optic tectum in both normal quail and chimera groups (Figure 9). These findings indicate that most quail axons follow a zebra-finch-like pattern of innervation in the zebra finch forebrain.

### Telencephalon sizes in chimeras are different

**Figure 10:**
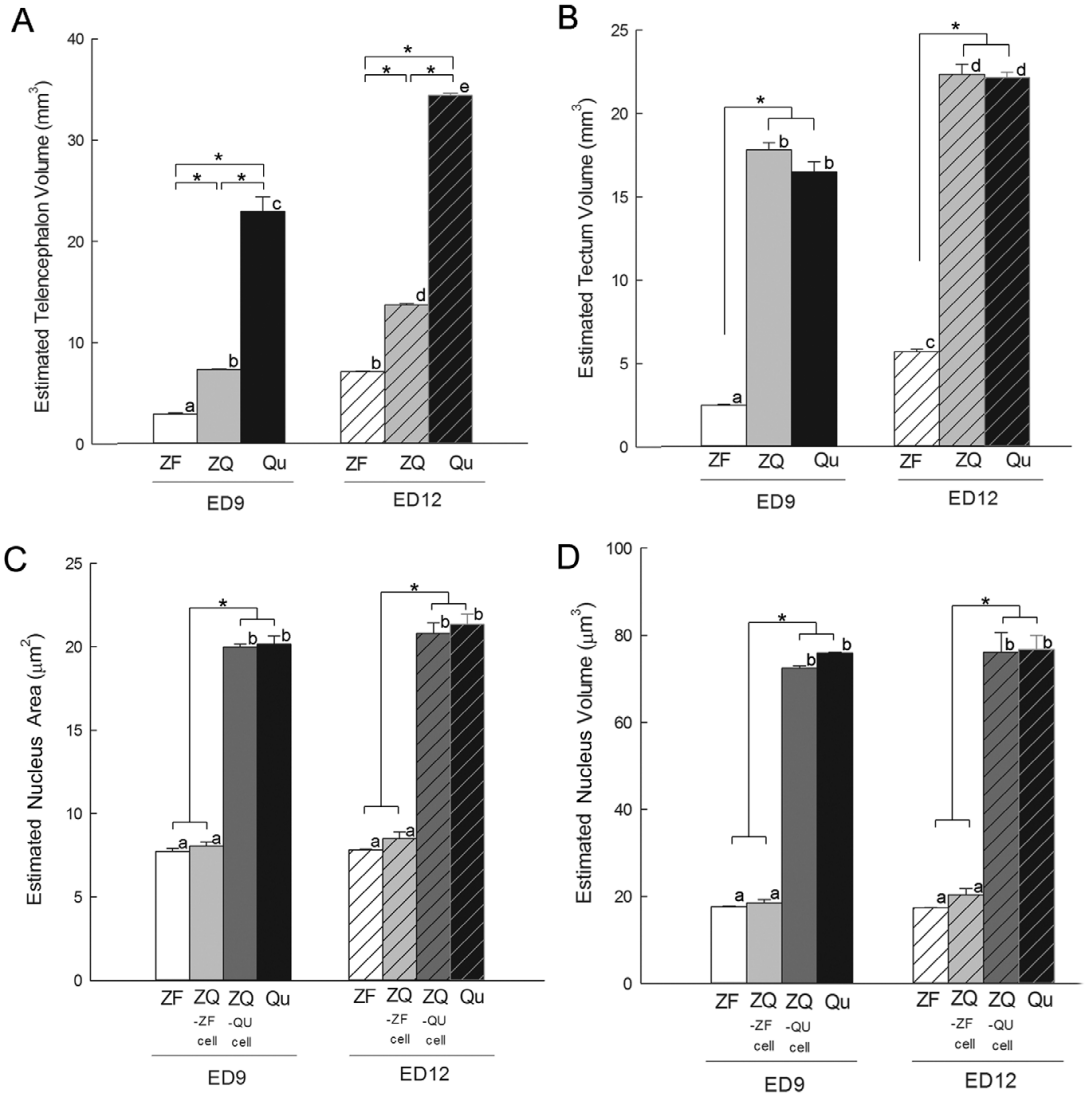
Sizes of telencephala, tecta and cell nuclei among zebra finches, chimeras and quails. (A) Estimated telencephalon volumes at ED9 and ED12. (B) Estimated tectum sizes. (C) Estimated cell nucleus areas. The cells of ZQ were measured from the zebra finch telencephalon and quail tectum in the chimeric embryos. (D) Estimated cell nucleus volumes. *: *p*<0.05, among groups; a–e: *p*<0.05 between stages; parametric one-way ANOVA, Tukey's post hoc test (at ED9, n = 4 ZF, 4 ZQ, and 3 QU; at ED12, n = 3 ZF, 3 ZQ, and 3 QU). Error bars, S.E.M.

As expected from their body size differences, the telencephalon volumes of normal zebra finches were significantly smaller than the telencephalon volumes of normal quails at ED9 or ED12 (Figure 10A). However, the telencephalon volumes of the zebra finch donor in zebra finch-quail forebrain chimeras were significantly larger than in normal zebra finches, but smaller than in normal quails (Figure 10A). In essence, the relative telencephalon size of the finch part of the chimeric brain at ED9 was similar to the volume of the normal zebra finch telencephalon at ED12 (Figure 10A). In contrast, the quail tectum sizes innervated by the zebra finch retinae of chimeras were similar to the tectum sizes of normal quail hosts at both developmental stages, and followed the quail developmental schedule (Figure 10B). Unlike this telencephalon size effect, the cross-species chimeras did not change representative cell nucleus areas (Figure 10C) or volumes (Figure 10D) of each species. These findings suggest that the quail host environment may have accelerated the developmental schedule of the zebra finch brain.

### Cell density and numbers are modified in the chimeras

**Figure 11:**
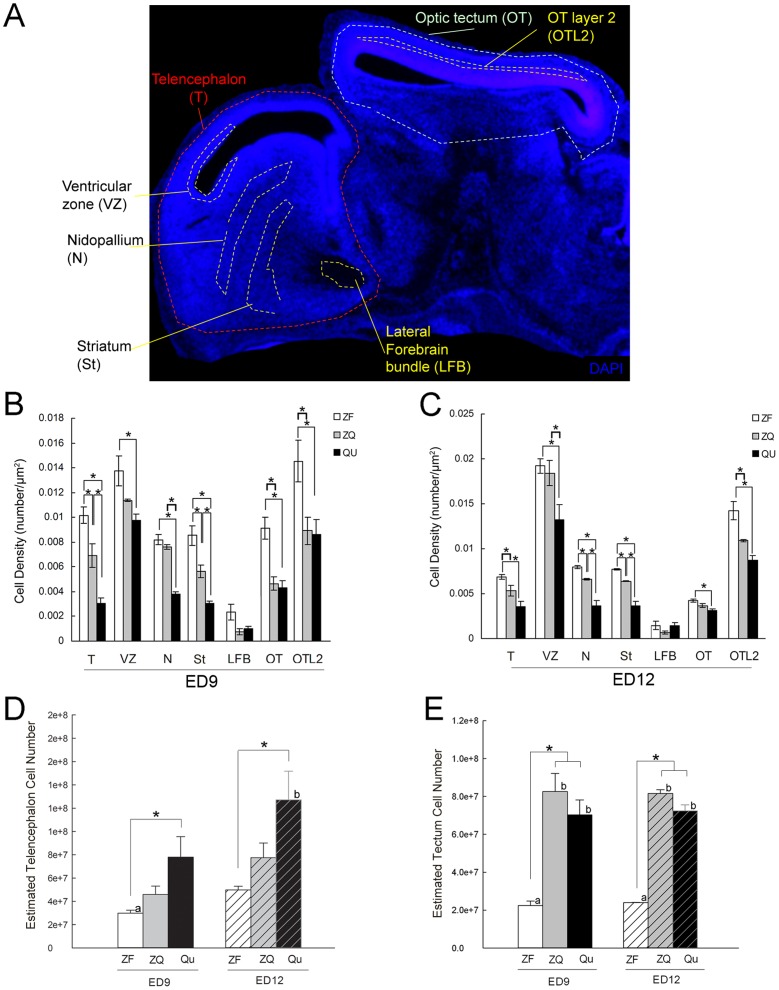
Quail host environment changes the cell density of the zebra finch telencephalon. (A) DAPI stained image of representative regions selected for cell density measurements in a quail sagittal sections at ED12. (B) Estimated cell density in subregions at ED9. (C) Estimated cell density in subregions at ED12. (D) Estimated total cell numbers in the telencephala at ED9 and ED12. (E) Estimated total cell numbers in the tecta. *: *p*<0.04, among groups; a–b: *p*<0.04 between stages; Kruskal–Wallis one-way ANOVA, Dunn's post hoc test in VZ in (B), St in (C), and in (D); parametric one-way ANOVA, Tukey's post hoc test in rest of regions in B, C and E (at ED9, n = 4 ZF, 4 ZQ, and 3 QU; at ED12, n = 3 ZF, 3 ZQ, and 3 QU). Error bars, S.E.M.

We stereologically analyzed DAPI-stained cell densities in chosen brain areas (Figure 11A). In the telencephalon (T), normal zebra finches had a higher cell density than normal quail (Figure 11B–C). In the chimeras, the finch telencephalic ventricular zone (VZ), which is the major source of forebrain neurogenesis [42], went from having a lower density similar to the quail at ED9 to a higher one similar to a normal zebra finch at ED12 (Figure 11B–C). Conversely, in pallial areas represented by the nidopallium (N), the cell density in the chimeras was similar to the normal zebra finch at ED9 (Figure 11B), but was lower than the normal zebra finch at ED12 (Figure 11C). In the striatum, the cell density of chimeras was between that of the zebra finch and quail at ED9 (Figure 11B) and ED12 (Figure 11C). We used the lateral forebrain bundle (LFB; containing few cell bodies) as a control region, and its densities were similar between species at both stages (Figure 11B–C).

In the optic tectum (all layers measured together), the cell density of the normal zebra finches was higher than that of quail at ED9, but subsequently diminished to near-quail levels by ED12. In the chimeras, although the quail tectal cell density was closer to that of normal quail at both ages (Figure 11B–C), it was not significantly different from normal finch values at ED12 (Figure 11C), suggestive of a small effect at ED12. To investigate whether this effect is specific to all layers of OT, we selected tectal layer 2 (OTL2), which contains more homogenous cell densities, and develops into the stratum griseum centrale (SGC), the source of the major OT efferent cells [43]. In this OTL2 layer, large differences remained at both developmental ages between normal zebra finches and quails, and the cell densities of chimeras were similar to the cell densities of normal quails (Figure 11B–C).

The total cell number in the telencephalon was estimated from average cell density and whole telencephalon size. We found that in normal embryos the total number of quail cells was much more than zebra finch cells at both ages (Figure 11D). However, in the chimeric zebra finch telencephala the total cell numbers were between the cell numbers of normal zebra finch and normal quail telencephala (Figure 11D). Interestingly, the quail tectal cell numbers in the chimeras were not significantly different from normal quails (Figure 11E). These findings indicate that the quail host had a significant effect on accelerating cell density changes in the zebra finch ventricular zone and telencephalon, and increasing the number of cells in the zebra finch telencephalon; but the finch donor only had a small influence on the cell density in the quail optic tectum.

### Proportions of telcenphalic brain subdivisions remain the same

**Figure 12:**
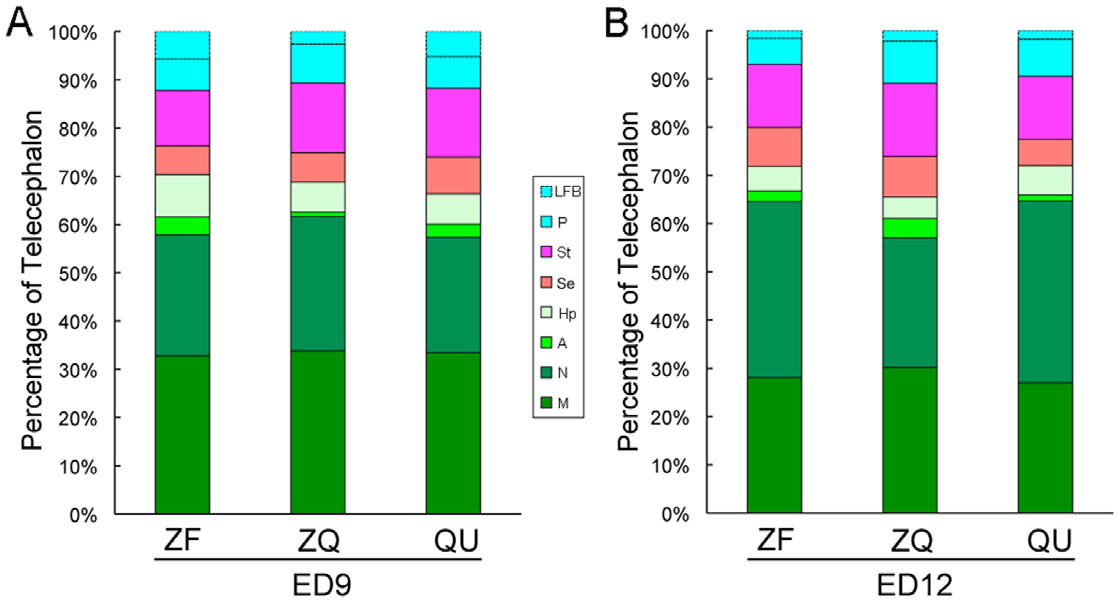
Proportions of telencephalon subregions in chimera at ED9 (A) and ED12 (B). There were no differences between groups; Kruskal–Wallis one-Way ANOVA, *p*>0.1 (at ED9, n = 4 ZF, 4 ZQ, and 3 QU; at ED12, n = 3 ZF, 3 ZQ, and 3 QU).

To determine whether the enlargement of the zebra finch chimeric telencephalon was proportional among subregions, we measured the size of telencephalic subregions and calculated their proportions by dividing their values by telencephalon size, calculated separately for normal zebra finch, chimeric, and normal quail embryos at the two developmental stages. We found that the proportional sizes of the lateral forebrain bundle (LFB), containing the fiber tracts that go between graft and host, showed no significant group differences at either ED9 (Figure 12A) or ED12 (Figure 12B). Likewise, the proportions of the zebra finch subpallium [pallidum (P), septum (Sp), and striatum (St)] and pallium [mesopallium (M), nidopallium (N), arcopallium (A), and hippocampus (Hp)] did not significantly differ among groups at either ED9 or ED12. Thus, the host quail environment did not change the relative proportions of the zebra finch telencephalic subregions.

## Discussion

Using avian cross-species *in ovo* transplantation of the neural tube at early embryonic stages, we substituted the forebrain between two species that diverged from a common ancestor more than 65 million years ago. After surgery, most developmental processes appeared to occur in the chimeras as they did in normal embryos. Both graft and host tissues maintained their species external morphological characteristics, similar to what is seen in transplants between more closely-related sister taxa like chick-quail and quail-duck chimeras [31], [44], [45]. These previous interspecies transplants have used embryos with similar developmental patterns (both precocial), unlike the zebra finches (altricial) and quail (precocial) embryos used here. In those studies there were no noticeable changes in brain sizes in the chimeras, although brain size differences exist between these species. However, in our study, the zebra finch forebrain in the chimeras attained a larger size at an earlier age, relative to a zebra-finch intrinsic schedule. This indicates that telencephalic enlargement in an altricial species is not a fully cell-autonomous process, but can be influenced by the developmental environment within the embryo.

In galliform birds, most neural projections (such as pallial-striatal projections) form during embryonic development [40], [41]. Such projections appeared to have formed in the zebra finch-quail forebrain chimeras. For example, the thalamic neurons and eyes from zebra finch grafts successfully projected to the spinal cord and tectum, respectively, of the quail host. The quail cranial nerve innervated the zebra finch upper beak. The quail host brain successfully projected to the zebra finch forebrain through fiber tracts of the lateral forebrain bundle. Interestingly, the quail innervation of the zebra finch telencephalon closely followed the zebra finch pattern. These findings suggest either that both distantly related species share a similar overall developmental pattern of neural connectivity with similar underlying mechanisms, or that where differences do exist, the “innervating" species cells are able to follow a trajectory set by the “innervated" species cells.

Chimeras with midbrain transplantation from quail donors into closely related chicken hosts can hatch, and such transplants transform the temporal pattern of crowing and auditory perceptual predisposition from a chicken host-like to a quail donor-like pattern [46], [47], [48]. Thus, it is possible that the grafted zebra finch telencephalon could attract quail host fibers according to a zebra finch pattern of connections, and could possibly function like a zebra finch forebrain. However, our chimera animals did not hatch. This could be because: 1) Species differences in developmental programs (altricial vs. precocial) result in a failure of coordination of the neural and non-neural changes that are generally prerequisite for hatching; 2) Incongruity in the sizes of the upper finch and lower quail beak may mechanically hinder the embryos from piercing the chorioallanotic membrane to commence air-breathing; or 3) The schedule for the commencement of air breathing in the chimera embryos may be disrupted by a mismatch between the developmental states in donor forebrain and brainstem host circuitry. Nevertheless, our findings support the idea that brain segments of donors and hosts can express their own local organizational principles, yet still conform to more global roles in behavioral circuit organization [49]. The “precocial" host embryo appears to have accelerated the schedule for size increases in the “altricial" donor embryo telencephalon.

In contrast to songbirds, galliform birds have a proportionally larger midbrain and hindbrain, including an expanded trigeminal system for feeding [2], [50]. These “enlarged" portions of the midbrain could be directly or indirectly involved in changing the volume of the telencephalon. For example, genetic manipulations of the size of thalamic inputs to the telencephalon in mice changed the sizes of primary sensory cortical areas innervated by the thalamus [51]. Molecules secreted from axons can influence telencephalic development by triggering gene expression changes, which lead to connectivity and structural changes [51], [52], [53]. This could be one possible explanation for why the zebra finch telencephala increased in size, by receiving direct or indirect innervation from the larger brainstem and midbrain areas of the quail host. An alternative view is that other factors cause changes in neurogenesis timing and cell cycle rates leading to species differences in telencephalon size [3], [10], [54]. Consistent with this idea, independent lineages of vocal learning species have a comparatively enlarged pool of progenitor cells in the subventricular zone [55]. Telencephalon enlargement in vocal learners could thus result from a larger “reserve pool" of proliferating cells. In the present study, cell densities in the normal zebra finch forebrain were higher than in quail throughout embryonic development, especially in the ventricular zone. Similarly, for mammals, one recent hypothesis for the expansion of cerebral cortex considers effects from both the neural-progenitor cell population and its local environment (for example, basal lamina and extracellular matrix proteins could influence the behavior of the progenitor cell population) [56], [57]. Our results show that in avian brain chimeras, cell density in the ventricular zone and other forebrain regions was significantly lower than in normal zebra finches, suggesting that the host environment altered one or more processes that influenced cell density. We suggest in this case that the host environment (including factors secreted from host brain or non-brain tissues) affected the local environment in the forebrain and modulated cell densities in the progenitor cell zone, changing donor forebrain size. It is also possible that a more global hormonal or hormone-like signal from outside the host embryo brain changed the characteristics of donor graft development, as seen in transplants involving non-neural tissues [58], [59]. A combination of these alternatives could also be at work.

The mechanisms proposed above are presumably not uniform across the brain. In the present study, normal quail tecta and host tecta in chimeras were similar in size, and had similar cell densities and cell numbers, in spite of the fact that in chimeric animals, smaller zebra finch retinae were connected to the quail tecta. Previous studies have suggested that the tectal size of avian species is related to the initial size of its corresponding progenitor region at the neural tube stage [10], [30], [60]. Taken together, we suggest that the size of the retinal ganglion cell population may not affect these aspects of tectal development in quail.

In summary, this study suggests that that the evolution of developmental telencephalic size changes between an altricial vocal-learning species and a precocial vocal non-learning species may depend on a mixture of cell-autonomous and cell-interdependent developmental processes. Further studies will be necessary to determine what cell-extrinsic factor(s) and intracellular signaling pathways are involved in these changes in telencephalic size development.

## Supporting Information

Figure S1 **A frontal view of dissected brain in chimeric head at ED16.** Optic nerves of zebra finch eyes innervate the quail optic tecta in the chimeric head.
(TIF)
