# Supplementary figures and images for: Interspecies Avian Brain Chimeras Reveal That Large Brain Size Differences Are Influenced by Cell–Interdependent Processes
Source: PLoS One. 2012 Jul 30;7(7):e42477. doi: 10.1371/journal.pone.0042477 (PMC3408455; doi:10.1371/journal.pone.0042477)

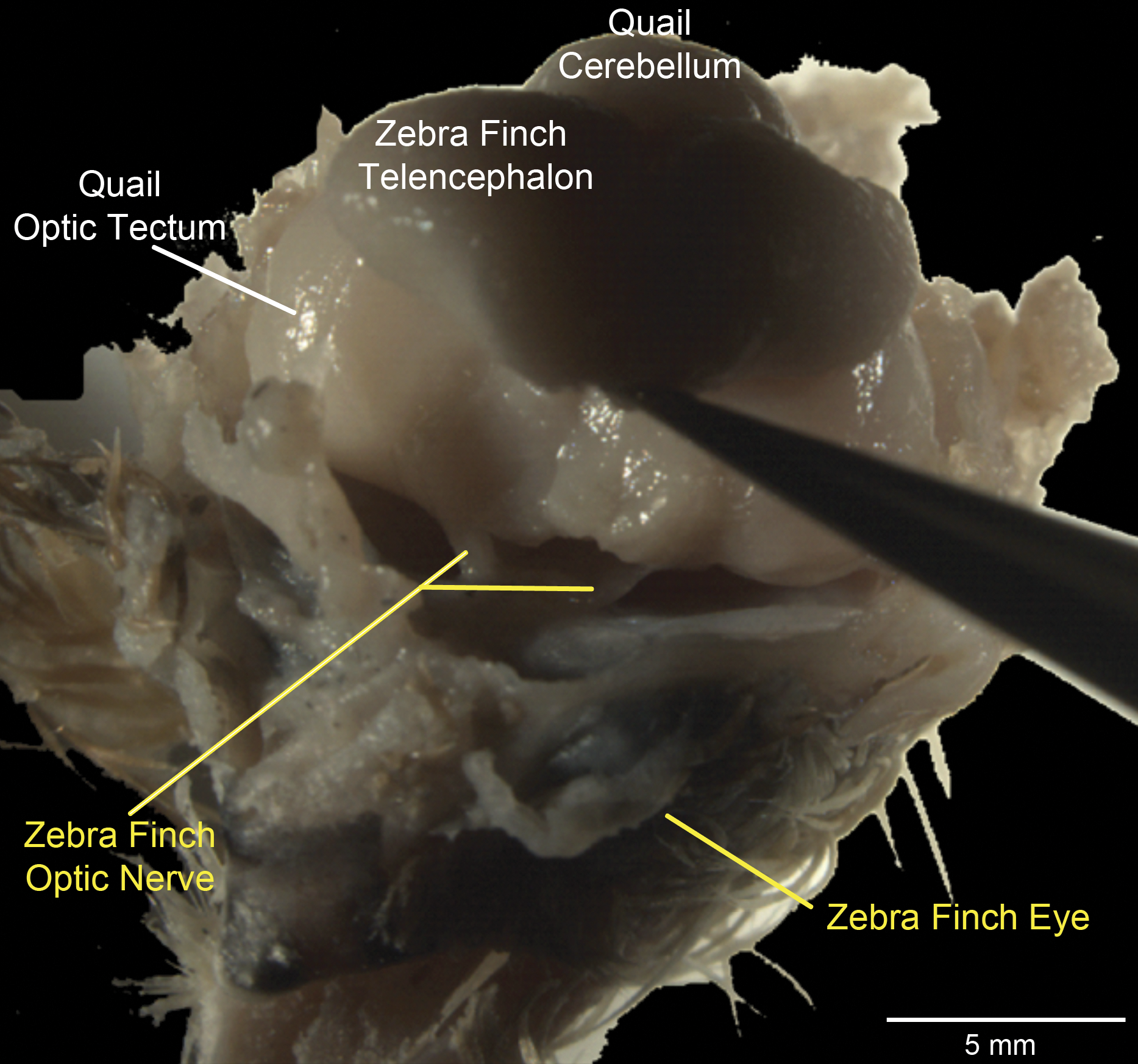

Supplement: Figure S1 — A frontal view of dissected brain in chimeric head at ED16. Optic nerves of zebra finch eyes innervate the quail optic tecta in the chimeric head. (TIF) [file pone.0042477.s001.tif]
